# Multiplexed Competitive Screening of One-Bead-One-Component Combinatorial Libraries Using a ClonePix 2 Colony Sorter

**DOI:** 10.3390/ijms20205119

**Published:** 2019-10-16

**Authors:** R. Ashton Lavoie, Alice di Fazio, Ruben G. Carbonell, Stefano Menegatti

**Affiliations:** 1 Department of Chemical and Biomolecular Engineering, North Carolina State University, Raleigh, NC 27695, USA; rabradl3@ncsu.edu (R.A.L.); abdifazi@ncsu.edu (A.d.F.); 2 Biomanufacturing Training and Education Center (BTEC), North Carolina State University, Raleigh, NC 27695, USA; 3 National Institute for Innovation in Manufacturing Biopharmaceuticals (NIIMBL), Newark, DE 19711, USA

**Keywords:** library screening, bioactive peptides, ligand development, affinity chromatography, affinity ligand, ClonePix 2

## Abstract

Screening solid-phase combinatorial libraries of bioactive compounds against fluorescently labeled target biomolecules is an established technology in ligand and drug discovery. Rarely, however, do screening methods include comprehensive strategies—beyond mere library blocking and competitive screening—to ensure binding selectivity of selected leads. This work presents a method for multiplexed solid-phase peptide library screening using a ClonePix 2 Colony Picker that integrates (i) orthogonal fluorescent labeling for positive selection against a target protein and negative selection against competitor species with (ii) semi-quantitative tracking of target vs. competitor binding for every library bead. The ClonePix 2 technology enables global at-a-glance evaluation and customization of the parameters for bead selection to ensure high affinity and selectivity of the isolated leads. A case study is presented by screening a peptide library against green-labeled human immunoglobulin G (IgG) and red-labeled host cell proteins (HCPs) using ClonePix 2 to select HCP-binding ligands for flow-through chromatography applications. Using this approach, 79 peptide ligand candidates (6.6% of the total number of ligands screened) were identified as potential HCP-selective ligands, enabling a potential rate of >3,000 library beads screened per hour.

## 1. Introduction

The screening of solid-phase combinatorial peptide libraries has found wide application in the development of biospecific agents, including ligands for the purification of biomolecules [1,2,3,4,5,6,7], diagnostic reagents [8,9], and medicinal compounds [10]. The recent expansion of peptide chemistry to non-natural amino acids, cyclic and polycyclic constructs, and peptidomimetics has spurred a strong interest in library screening for the discovery of bioactive ingredients [10,11]. A myriad of approaches and instruments have been introduced to increase throughput and decrease the frequency of false positives. Notable examples include the expansion of selection criteria in the COPAS Flow Pilot system [3,4,12], the Pickoscreen confocal nanoscanning and bead-picking platform (CONA) [13], library screening via microfluidic sorting [14], bead blot approaches [15], methods to reduce interference from non-specific binding [16,17], and the emergence of low-cost automated systems for library screening by wide-field fluorescence microscopy [18]. While great progress has been achieved in throughput and the ability to modulate the binding strength of the identified leads to match target specifications, current techniques rarely focus on tailoring binding selectivity based on the complexity of the intended application. This aspect is particularly relevant in diagnostic applications or the recovery of rare species, where the target molecule is present at a much lower concentration relative to other species. Another application that poses strong requirements on binding selectivity is the development of ligands that operate in negative binding mode (i.e., capture all the competing species without binding the target molecule) for use in flow-through chromatography or enrichment of low abundance species for mass spectrometry [19,20]. In this case, the simultaneous positive/negative screening increases the complexity of the system and prevents the use of multichannel approaches to minimize signal-to-noise ratio, as discussed by Hintersteiner et al. [12]. However, because applications in negative binding mode are highly diverse in terms of products and contaminant populations, and minimal product binding is the only requirement to be stringently enforced, they allow for a more lenient approach to be adopted towards false positives.

The polydispersity between library beads and the diversity of proteins present major challenges for fluorescence-based library screening in the form of variable (i) polymer bead autofluorescence, (ii) fluorescence intensity of the target biomolecule, and (iii) fluorescence intensity of the ensemble of contaminant proteins. These challenges pose the need for a novel technology that can survey the range of bead aspect ratios and fluorescent signals for both target and contaminants across the whole pool of library beads prior to screening, and utilize this information to tailor the selection criteria of average bead fluorescence intensity and ratio of target vs. contaminant fluorescence intensity. This approach eliminates outliers, minimizes the loss of false negatives, and greatly promotes the selection of beads carrying candidate peptide ligands with desired binding strength and specificity. Ensemble imaging of combinatorial libraries followed by informed selection of beads with fluorescent intensity adjusted to the remaining population has been successfully demonstrated by Heusermann et al. [18] This system to select beads using wide-field fluorescence microscopy allows for high-throughput bead selection; in its current version, however, it requires bead picking to be manually operated. To expand on this “cast-image-pick” approach, this work adapted a ClonePix 2 colony picker (Molecular Devices, Sunnyvale, CA) to fully automate the bead picking process with minimal operator intervention. The ClonePix 2 enables distribution-driven thresholding of the whole ensemble of beads to account for the bead size and average peptide density, chosen fluorescent tags, target vs. contaminants ratio, and screening conditions. By segregating the beads, the machine allows for image characterization interference in intensity measurements from near neighbors, thereby strengthening the accuracy of population-based sorting criteria. Furthermore, the ClonePix 2 incorporates a stringent wash step of the “pick head”, the apparatus used to aspirate the target from the semi-solid matrix to prevent carry-over during selection.

In this work, we demonstrate the use of ClonePix 2 for library screening by selecting peptide ligands for protein purification by flow-through mode chromatography. To this end, we designed a multiplexed dual-fluorescence screening approach to identify an ensemble of peptides that cooperatively target a set of protein contaminants, while showing no affinity for the molecule to be enriched. In this study, we adopted a model screening feed containing >300 host cell proteins (HCPs) from a null (i.e., non-producing) Chinese hamster ovary (CHO) cell culture harvest [19] as protein contaminants and human polyclonal immunoglobulin G (IgG) as target proteins. The CHO HCPs were collectively labeled with a fluorescent tag and IgG was individually labeled with an orthogonal fluorescent tag. A synthetic one-bead-one-peptide (OBOP) library with focused amino acid composition was incubated with labeled HCPs and IgG, washed and cast into a methylcellulose-based semi-solid matrix (CloneMatrix), and finally imaged and picked based on gate criteria set up for the full population screened. The sequences carried by the selected beads were identified by mass spectrometry and individually conjugated to chromatographic Toyopearl resin. The resulting peptide-based adsorbents have been characterized in published work [19], demonstrating that the selected peptide ligands effectively capture the whole spectrum of CHO HCP contaminants, while allowing the IgG product to flow through unbound. While this work demonstrates a selection of peptides against protein targets and competitors, this approach can be broadly applied to any target/ligand system where the target can be fluorescently labeled and the ligands are available as an one bead one component (OBOC) solid-phase library.

## 2. Results and Discussion

The identification of an ensemble of ligands for negative chromatography represents a paradigmatic case study of multiplexed library screening. The mixture of Chinese hamster ovary (CHO) host cell proteins (HCPs) and human IgG in a 1:4 weight ratio used as model feedstock in this study has been characterized in previous work [19]. This feedstock mimics the cell culture harvests produced in biopharmaceutical processes, and is ideal for the identification of peptide ligands that operate in negative binding more readily, that is, when immobilized on a chromatographic substrate, selectively capturing HCPs (impurities) while letting the IgG (product) flow through unbound [19].

**Figure 1:**
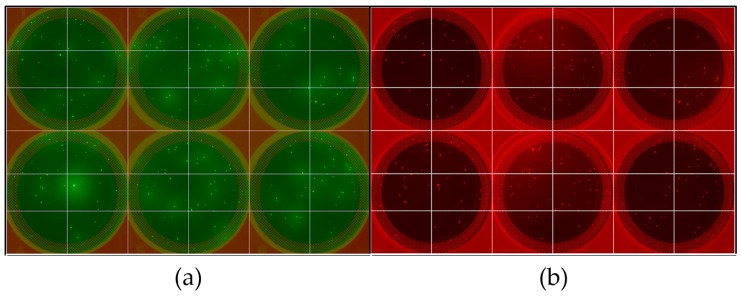
Fluorescence imaging by ClonePix 2 of unbiased combinatorial linear peptide library on HMBA-ChemMatrix resin after incubation with fluorescently tagged immunoglobulin G (IgG) and CHO-S host cell proteins (HCP). (**a**) Library is imaged with a ClonePix 2 FITC filter to visualize beads bound to IgG tagged with Alexa Fluor 488. (**b**) The same plate is imaged with a ClonePix 2 Rhodamine filter to visualize beads bound to CHO HCP tagged with Alexa Fluor 546.

A hexametric combinatorial OBOP (one-bead-one-peptide) library X1-X2-X3-X4-X5-X6-G-S-G was first synthesized on HMBA-ChemMatrix resin via Fmoc/tBu synthesis following the “split-couple-and-recombine” method proposed by Lam et al. [19,20]. Specifically, a limited library of ~400,000 beads (out of 1,000,000 possible sequences) was synthesized as described in [19,20] by filling the six combinatorial positions X1-X6 using isoleucine (I), alanine (A), glycine (G), tyrosine (Y), aspartate (D), histidine (H), arginine (R), lysine (K), serine (S), and glutamine (Q). The Glycine-Serine-Glycine C-terminal spacer (G-S-G) was introduced to aid in post-selection sequencing of the selected leads. ChemMatrix was chosen as the resin for library synthesis and screening, as it is resistant to a wide range of reagents and solvents, its hydrophilic surface minimizes non-specific protein binding, and its translucency makes it ideal for fluorescence-based selection [3]. The hydroxymethylbenzoic acid (HMBA) linker enables cleavage of the peptides from the ChemMatrix solid phase, thus allowing for sequencing of the peptides carried by the selected beads by liquid chromatography tandem mass spectrometry (LC/MS/MS). The CHO HCPs were fluorescently labeled with red Alexa Fluor 546 (AF546) NHS ester label, while human IgG was labeled with Alexa Fluor 488 (AF488) NHS ester label. Following deprotection, an aliquot of the library (~24,000 beads, 0.1 mL settled volume) was equilibrated in phosphate buffered saline, pH 7.4, 0.1% Tween 20 (PBS-T), and incubated with a ~1:3 mixture of red-HCPs and green-IgG in PBS-T. After incubation, the library beads were washed and gently suspended in 1X CloneMatrix semi-solid media prepared in PBS pH 7.4 + 0.1% Tween 20, then cast in 6-well polystyrene plates (20-25 beads per well). The plates were then imaged by fluorescence microscopy on the ClonePix 2 instrument using the FITC and Rhodamine filter lines to respectively detect AF488 and AF546 (Figure 1).

While initially developed by Molecular Devices to conduct high-throughput screening of mammalian cells, the ClonePix 2 colony picker is amenable to OBOC screening. The ClonePix 2 system, in fact, can fully automate bead imaging and selection, while requiring minimal modifications to process ChemMatrix beads, whose diameter (~100–300 µm) resembles that of cell colonies. To ensure that the polymers present in the CloneMatrix do not interfere with the subsequent peptide sequencing by LC/MS/MS by reduction of the peptide MS signal, beads carrying a known sequence (HWRGWVGSG) were sequenced pre- and post-casting in CloneMatrix, with no impact on sequencing. In addition to the library beads, the sequence GSGSGSGSG, known to have minimal interaction with the model proteins utilized in this work, was also synthesized on HMBA-ChemMatrix and incubated with the fluorescent proteins as a negative control.

**Figure 2:**
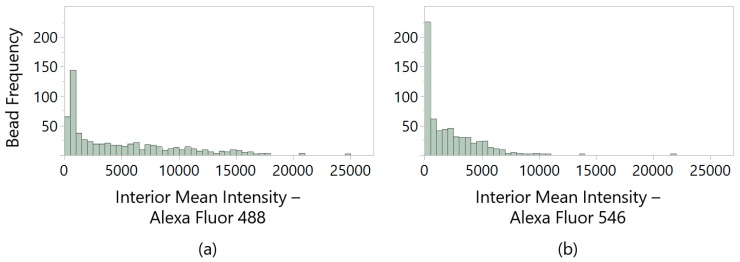
Distribution of interior mean intensities for a one-bead-one-compound combinatorial peptide library incubated with Alexa Fluor 488-labeled polyclonal human immunoglobulin G (IgG) and Alexa Fluor 546-labeled CHO-S host cell proteins (HCP). (**a**) shows the interior mean fluorescent intensity with the ClonePix 2 FITC filter at 800 ms exposure to detect the labeled IgG. (**b**) shows the detected distribution from the ClonePix 2 Rhodamine filter at 500 ms exposure for HCP detection. Interior mean intensities were calculated from imaging data collected by the ClonePix 2 software for all image features not excluded as irregular by the software algorithm.

To select ligands that selectively bind the whole spectrum of HCP proteins, we established gates for a maximum allowable AF488 intensity and a minimum AF546 intensity detected as measures of IgG and HCP capture, respectively. These pick gates were based on the distribution of interior mean fluorescent intensity (Figure 2a) calculated using the ClonePix 2 imaging software, wherein “interior mean intensity” is defined as the arithmetic mean within the detected bead radius. Beads were excluded when they exceeded the interior mean intensity gate for AF488 (i.e., were identified as IgG-binding) at the geometric mean of all beads imaged (geometric mean = 2417.7, rounded to 2500). Because HMBA-ChemMatrix beads show intrinsic fluorescence in this range (the arithmetic mean of GSGSGSGSG negative control beads was measured at 755.6 ± 380.0), we imposed a higher exclusion gate criterion to distinguish IgG-binding beads from bare beads. Of the beads remaining after IgG-binding gating, a secondary criterion was set based on the bead radius to exclude visible features on the plates that either were unlikely to be beads or included multiple yet undistinguished beads (clusters).

For detection of HCP-bound beads, the geometric mean for interior mean intensity from imaging with the ClonePix 2 Rhodamine filter (detection of AF546-HCPs) was measured at 620.1 (Figure 2b) with an arithmetic mean of 407.9 ± 158.1 for GSGSGSGSG negative control beads. In this case, however, the gate criteria were set loosely at the exclusion of beads with an interior mean intensity <100 for automated bead picking. Further gating using the geometric mean of HCP intensity from the library at 620 was imposed for post-automated bead picking. All beads selected for automated picking by the ClonePix 2 system were transferred one bead per well into 96-well plates pre-loaded with PBS.

To determine whether all beads selected were effectively deposited, the 96-well plates were visually inspected to confirm the presence or absence of a single bead in each designated well. Plate transfer efficiency, defined as the ratio of the number of wells confirmed to be occupied by a bead divided by the number of beads transferred, was measured by imaging the resulting 96-well plates using the Molecular Devices CSI-FL fluorescent imager; the percentage of occupied wells that had more than one bead deposited was also monitored. The plate transfer efficiency was calculated at 71% ± 5.9%, with 14% ± 16% of occupied wells carrying more than one bead. Bead transfer efficiency can be improved by optimizing matrix curing to obtain a more rigid support during picking, or adjusting the parameters for bead aspiration and depositing.

**Figure 3:**
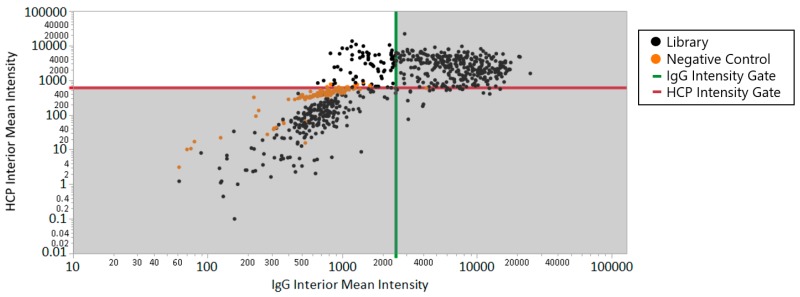
Immunoglobulin G (IgG) Interior Mean Intensity vs. Host Cell Protein (HCP) Interior Mean Intensity of limited library of X1-X2-X3-X4-X5-X6-G-S-G one bead one component (OBOC) peptide library incubated with Alexa Fluor 488-labeled polyclonal IgG and Alexa Fluor 546-labeled CHO-S clarified harvest host cell proteins. The pick gates at <2500 FITC interior mean intensity for IgG and >620 Rhodamine interior mean intensity for HCP are represented by the green and red reference lines, respectively. The highlighted region in the top left quadrant represents lead HCP-specific bead candidates.

The comparison between the beads selected for specific binding of HCPs and the full library is shown in Figure 3, where the upper left quadrant includes the beads that met both the HCP intensity criteria (>620) and IgG intensity criteria (<2500), corresponding to 79 positive beads out of 1189 total bead-like features detected (6.6% positive hits). The lead beads from this work were sequenced and their HCP binding properties were characterized in detail in published work [19]. Most notably, the selected HCP-binding peptides showed considerable sequence homology, grouped in multipolar and hydrophobic positive sequences [19], and afforded specific capture of HCPs with minimal binding of IgG. While positive controls were not explicitly analyzed with the ClonePix 2 platform in this work, the selection of an antibody-binding peptide (HWRGWV) from a mixture of peptide-functionalized beads (HWRGWV-ChemMatrix and a portion of a combinatorial OBOP library) has previously been demonstrated using this multiplexed approach implemented on a microfluidic bead-sorting platform [14].

The throughput of the “cast-image-pick” strategy presented here is heavily dependent on the gate criteria used for selecting positive beads. In this workflow, the main bottleneck is represented by the total number of beads to pick as opposed to the total number of beads in the library screened. While the ClonePix 2 instrument is capable of picking beads on the order of 200 positive beads picked per hour [21], the gate criteria applied to our system (resulting in 6.6% positive beads picked) would allow for >3000 total beads screened per hour when considering all beads imaged rather than individual beads picked.

## 3. Materials and Methods

### 3.1. Materials

HMBA-ChemMatrix resin, 1,2-ethanedithiol (EDT), and triisopropylsilane (TIPS) were sourced from MilliporeSigma (St. Louis, MO, USA). N′, N′-dimethylformamide (DMF), methanol, N-methyl-2-pyrrolidone (NMP), and dichloromethane (DCM) were sourced from Fisher Chemical (Hampton, NH, USA). Fmoc/tBu-amino acids, O-(7-Azabenzotriazol-1-yl)-N,N,N′,N′-tetramethyluronium hexafluorophosphate (HATU), diisopropylethylamine (DIPEA), piperidine, and trifluoroacetic acid (TFA) were sourced from Chem-Impex International (Wood Dale, IL, USA). CloneMatrix semi-solid media was generously donated by Molecular Devices (Sunnyvale, CA, USA). Lyophilized human polyclonal antibody was obtained from Athens Research (Athens, GA, USA). 0.2 µm bottle top filters, N-methylpyrrolidone (NMP), and Grenier Bio-One 6-well plates were obtained from VWR International (Radnor, PA, USA). Sodium phosphate (monobasic), and Tween 20 3 kDa MWCO Amicon filters were obtained from MilliporeSigma (St. Louis, MO, USA). Sodium chloride, sodium phosphate (dibasic), sodium hydroxide, and hydrochloric acid were obtained from Fisher Chemical (Hampton, NH, USA). CHO-S null cell culture harvest was provided by the Biomanufacturing Training and Education Center (BTEC) at NC State University. Macrosep Advance 3 kDa MWCO centrifugal filters were obtained from Pall Corporation (Ann Arbor, MI, USA).

### 3.2. Methods

#### 3.2.1. Solid Phase Peptide Synthesis and Deprotection

The solid-phase peptide libraries, and HWRGWVGSG and GSGSGSGSG control peptides were synthesized on HMBA-ChemMatrix (loading = 0.6 mmol/g) by Fmoc/tBu strategy on a Syro II automated peptide synthesizer (Biotage) as described in detail in prior work [19,20]. Briefly, bare resin was aliquoted into 100 mg aliquots and swelled in DMF at 40 °C for 20 min. Couplings for all amino acids were performed at a 3- to 5-molar excess of Fmoc-amino acids to the resin loading. A 1:1:4 Fmoc-amino acid:HATU:DIPEA molar ratio was used for coupling, with DIPEA solubilized in NMP. Couplings proceeded at 45 °C for 20 min with intermediate vortexing. Three to four couplings were employed per amino acid cycle prior to Fmoc deprotection to maximize reaction completion. Fmoc-deprotection was performed post-DMF wash at 20% piperidine for 20 min at room temperature. For the combinatorial library, a split-couple-recombine method [22] was employed to achieve an OBOC solid-phase library. Post-synthesis, resins were washed five times with ~10 mL DMF, then DCM and dried to a fine powder under nitrogen sparge. Side-chain deprotection was then performed via incubation with 94% TFA, 1% EDT, 3% TIPS, 2% deionized water solution for 2 h. Resins were then washed with DMF and 20% methanol, then stored in 20% methanol at 2–8 °C.

#### 3.2.2. Fluorescent Labeling of IgG and CHO-S HCPs

The CHO-S cell culture harvest [19] was clarified via centrifugation at 8000× *g* for 30 min then filtered with a 0.2 µm PES membrane using VWR Full Assembly Bottle-Top vacuum filters, followed by concentration to 2.3 g/L and diafiltration into 50 mM sodium phosphate, 20 mM sodium chloride, pH 8.3 using Macrosep Advance 3 kDa Centrifugal Filters. Human polyclonal IgG was dissolved in 50 mM sodium phosphate, 20 mM sodium chloride, pH 8.3 at 5 g/L. Alexa Fluor 546 and Alexa Fluor 488 were dissolved at 1 mg/100 µL extra dry DMF and immediately combined with 1 mL of the diafiltered harvest for AF546 (HCP-AF546) and 1 mL IgG for the AF488 (IgG-AF488) and incubated on a rotator at room temperature and light protected for 1 hour. Each sample was then diafiltered into PBS, pH 7.4 using Amicon Ultra 3 kDa MWCO filters to remove unreacted dye.

#### 3.2.3. Fluorescence Screening of Solid Phase Peptide Libraries Against IgG and CHO-S HCPs

Following equilibration in PBS, pH 7.4, the deprotected library (~100 µL) and control resins (~20 µL) were individually mixed with the labeled proteins in 0.2% Tween 20 in PBS, added to obtain a final concentration of 1.3 mg/mL IgG-AF488 and 0.58 mg/mL HCP-AF546, and incubated overnight at 2–8 °C. Resin beads were then washed with 0.1% Tween 20 in pH 7.4 (PBS-T) and suspended in a semi-solid CloneMatrix solution. The matrix was prepared from two parts Molecular Devices CloneMatrix and three parts 83.3 mM sodium phosphate, 250 mM NaCl, 0.17% Tween 20. Aliquots of 5 to 10 µL of settled library beads were gently incorporated into the matrix solution, and evenly aliquoted on a 6-well plate. The plates were then incubated at 37 °C for 2–18 h to cure the matrix. A ClonePix 2 colony picker (Molecular Devices in Sunnyvale, CA) was used for fluorescent imaging and selection of library and control beads. Specifically, the plates were imaged using the ClonePix FITC (800 ms exposure, 128 LED intensity) and Rhod (500 ms, 128 LED intensity) laser lines to monitor AF488 and AF546, respectively. Due to slight autofluorescence of the ChemMatrix beads under the FITC filter, bead location (i.e., ClonePix 2 run “Prime Configuration”) was assigned based on fluorescence intensity from the FITC filter. Beads were picked for further processing based on the following characteristics: (i) FITC interior mean intensity <2500, (ii) Rhodamine interior mean intensity >100, (iii) 0.05–0.25 mm radius. Picking was performed in suspension mode, with 20 µL aspiration volume to pick up the bead, and a 60 µL expel volume (the excess volume above the aspirated liquid was water). Picked beads were further gated at >620 Rhodamine interior mean intensity for subsequent sequencing.

## 4. Patents

Menegatti, Stefano; Lavoie, R. Ashton; di Fazio, Alice; Carbonell, Ruben G. Peptide Ligands for Capture of Host Cell Proteins. U.S. Provisional Patent Application No. 62/784,104, 21 December 2018.

## Abbreviations

NHS	N-hydroxysucciinimide
HMBA	Hydroxymethylbenzoic acid
SPPS	Solid phase peptide synthesis
LC/MS/MS	Liquid chromatography tandem mass spectrometry
Q-ToF	Quadrupole-time of flight
DMF	N′,N′-Dimethylformamide
Fmoc	Fluorenylmethyloxycarbonyl
HATU	O-(7-Azabenzotriazol-1-yl)-N,N,N′,N′-tetramethyluronium hexafluorophosphate
DIPEA	Diisopropylethylamine
NMP	N-methyl-2-pyrrolidone
TFA	Trifluoroacetic acid
EDT	ethanedithiol
TIPS	triisopropylsilane
